# Investigating the effects of peptide-based, MOS and protease feed additives on the growth performance and fecal microbial composition of weaned pigs

**DOI:** 10.1186/s40104-022-00681-8

**Published:** 2022-03-17

**Authors:** Prakash Poudel, Ryan Samuel, Crystal Levesque, Benoit St-Pierre

**Affiliations:** 1 Current address: Himalayan Pet Foods, Mukilteo, Washington, 98275 USA; 2 Department of Animal Science, South Dakota State University, Animal Science Complex, Box 2170, Brookings, SD 57007 USA

**Keywords:** Bacteria, Gut, Microbiome, MOS, Peptide, Protease, Swine, Weaning

## Abstract

**Background:**

Digestive disorders in weaning pigs remain a major challenge for swine producers. Different types of commercial feed additives have been developed to promote gut health and development in young pigs, but their effects on resident gut microbial communities remain largely unexplored. The aim of this study was to investigate the impact of a peptide-based product (Peptiva) in combination with mannose oligosaccharides (MOS) and an exogenous protease on the performance and fecal microbiome of nursery pigs.

**Methods:**

A total of 1097 weaned pigs were divided into 44 pens (24–26 pigs/pen) with each pen randomly assigned to one of four experimental diets as part of Phase II and Phase III of a standard nursery phase feeding program. Fecal samples collected from representative control and treatment pigs were used to investigate bacterial composition profiles by high throughput sequencing of PCR-generated amplicons targeting the V1-V3 region of the 16S rRNA gene.

**Results:**

Higher gain:feed was observed for pigs fed Peptiva and MOS compared to Controls during the period when experimental diets were fed, but the benefits of supplementation were not maintained after pigs were transitioned to a non-supplemented diet. Three candidate bacterial species, identified as Operational Taxonomic Units (OTUs), were found to have significantly different abundances between control samples and treatment samples during the same phase. In Phase III samples, SD_Ssd-00039, predicted to be a strain of *Streptococcus alactolyticus* based on nucleotide sequence identity, was the most highly represented of these OTUs with an average abundance in pigs fed Peptiva, MOS and protease that was 3.9 times higher than in Controls. The report also presents evidence of microbial succession that occurred during the trial, with 16 of the 32 most abundant OTUs found to vary between Phase II and Phase III samples for the same dietary treatment.

**Conclusions:**

Dietary supplementation with a combination of a peptide-based product, MOS, and protease increased the growth performance of weaned pigs compared to control animals during the nursery phase, but these benefits were no longer observed within 2 weeks after all animals were transitioned to a non-supplemented diet. Supplementation with these feed additives was found to modulate the composition of the swine gut microbiome during this period.

**Supplementary Information:**

The online version contains supplementary material available at 10.1186/s40104-022-00681-8.

## Introduction

Weaning is a critical stage during the swine production cycle, as it involves dramatic changes that affect the development of young animals and their overall health, which in turn directly impact their future performance and profitability [1]. Under typical intensive production conditions, weaning is associated with a number of abrupt changes, such as separation anxiety from the dam, adaptation to an unfamiliar physical environment and a new social hierarchy, as well as the transition to a different diet [2, 3]. While each of these changes represents an important stressor, the sudden switch in diet has a more direct impact on gut physiology. Indeed, the transition from milk, a highly digestible and palatable source of nutrients, to dry feed consisting mostly of plant-based ingredients requires physiological adaptations in digestive enzyme activities and gut secretions [2, 4, 5]. For instance, brush border digestive enzyme activities are lower after weaning [6], and there is a significant reduction in pancreatic secretions of trypsin, chymotrypsin, and amylase [7, 8]. Weaning induces both acute and long lasting structural and functional changes in the small intestine, such as shortening of villi and an increase in crypt depth [2, 6, 9]. These structural and functional adjustments of the gastrointestinal tract not only impact digestive and secretory functions, but also nutrient absorption capacity and barrier functions. Consequently, weaning is typically associated with a reduction in feed intake, but these changes can have more profound negative impacts on gut function and health. Under these conditions, exacerbated by other factors such as low immune protection, there is an increased susceptibility to enteric pathogens and other disorders that may lead to post-weaning diarrhea. Post-weaning diarrhea reduces feed conversion and growth of affected animals, and it can increase morbidity and mortality rates, ultimately resulting in economic losses for producers [5, 6, 10].

Impaired host digestion and absorption in the small intestine during weaning provide increased availability of nutrients for microbial opportunists, increasing the risk of gut dysbiosis and setting the stage for pathogen colonization and proliferation [11–13]. For instance, bacterial fermentation of undigested dietary proteins can result in increased concentrations of short chain fatty acids (SCFA) and of nitrogen-containing end-products, such as ammonia and amines, which can induce diarrhea [14–16]. Increased production of nitrogen end-products from gut microorganisms is not only detrimental to gut health, but it is also a concern for the environment [12, 17]. It is then a common practice to provide easily digestible protein sources, such as fishmeal, to pigs in the early stages of the weaning transition. Since the cost of such protein sources are becoming increasingly prohibitive, there is a strong incentive to transition from animal protein to more cost-effective plant protein sources, such as soybean meal, as early as possible. However, plant-based protein ingredients present a number of challenges for swine diets, such as the presence of plant fiber and anti-nutritional factors, as well as amino acid profiles that may be not be optimal for growth [18, 19].

In this context, strategies have been designed to promote swine gut development and functional maturation, as well as gut health, after weaning while mitigating the negative effects of plant-based protein sources. Nutrient availability from plant-based sources can be improved with dietary supplementation of exogenous enzyme preparations containing α-amylase, β-mannanase, xylanase, phytase, and/or cellulose to digest plant polysaccharides. Their addition to swine and poultry diets has indeed been shown to be beneficial [19–22]. Supplementation with exogenous proteases has also been found to help, as they can break down anti-nutritional factors [23, 24] or starch bound proteins, thus improving digestibility and nutrient availability [25]. In weaned pigs, the use of exogenous enzymes has been reported to benefit growth rate, nutrient digestibility, intestinal development, as well as host pepsin, pancreatic amylase and trypsin activities, while reducing fecal NH_3_ emissions [12, 24, 26, 27].

Other approaches to improve swine gut development, function, and health during weaning include supplementing diets with prebiotics such as mannose oligosaccharides (MOS). MOS consist of branched carbohydrates that are most commonly derived from the cell wall of *Saccharomyces cerevisiae* [28]. MOS have been reported as a viable alternative to antibiotics as well as a potent growth promotor when fed to pigs, with a number of studies showing that their addition to swine diets can increase performance metrics such as average daily gain, feed efficiency, and weaning weight [29–32].

More recently, peptide supplements have been reported as another effective strategy to promote gut function and health in weaned pigs. Depending on the source and the processing methods used, certain types of peptides have been found to be more than just a source of amino acids, as they can modulate biological activities affecting neural, endocrine, immune, and antioxidant functions, as well as enhance mineral availability and absorption [33–36]. Certain peptides have shown anti-viral properties as well as anti-microbial effects against a broad spectrum of bacteria and fungi [37]. Notably, supplementation of pig diets with anti-microbial peptides was reported to have positive effects on performance, nutrient digestibility, intestinal morphology, immune function, as well as intestinal microbiota [38–40]. Indeed, weaned pigs fed antimicrobial peptides such as AMP-A3, AMP-A5, colicin A1, cecropin AD, cipB-lactoferricin-lactoferrampin, defensin, and plectasin not only showed lower incidence of post-weaning diarrhea, but also enhancement of growth performance and improved nutrient digestibility [41–45].

In light of the importance of the gut microbiome in maintaining the health of the animal host and contributing to its nutrition [46], the beneficial effects of feed additives and exogenous enzymes on swine performance are likely to involve changes in the composition of symbiotic microbial communities. While great progress has been made in this field, the available information on the effects of combining different feed additives on the gut microbiome of pigs during weaning remains limited. In this context, the current report describes a study on supplementing swine diets with a combination of a commercial peptide product and MOS, with or without a protease, during the weaning phase. Combining the three additives was found to benefit feed efficiency in weaned pigs, and three candidate bacterial species identified as OTUs were found to differ in abundance in supplemented animals compared to control. Evidence in support of microbial succession occurring during the experimental period is also presented.

## Materials and methods

### Animals and dietary treatments

The animal trial was conducted at the South Dakota State University (SDSU) On-Site Wean-to-Finish Barn, with all procedures approved by the SDSU Institutional Animal Care and Use Committee before the start of the study. A total of 1097 weaned pigs (~ 7 kg; 21 days of age; blocked by weight), representing an equal mix of gilts and barrows from the DNA 610 genetic line, were randomly divided into 44 pens (24–26 pigs/pen; pen dimensions: 3.1 m × 6.9 m), with each pen randomly assigned to one of four experimental diets (Table 1). Pigs were fed a standard nursery phase feeding program: Phase I (d 1–7), Phase II (d 8–21) and Phase III (d 22–35). Phase I was a starter diet common to all pigs on trial, with experimental diets implemented during Phase II and Phase III. After completion of Phase III, pigs from all treatments were fed the same non-supplemented diet as the controls for a period of 2 weeks (d 36–49). At the end of the trial (d 49), 1076 pigs remained, as 21 pigs were removed due to illness, inability to thrive or death.

**Table 1:** Formulation and nutrient composition of experimental diets

Item^a^	Phase II				Phase III			
	Con	PMP^**b**^	PMP90^**c**^	PM^**d**^	Con	PMP^**b**^	PMP90^**c**^	PM^**d**^
Corn	662.5	651.5	672.2	652.2	953.10	942.1	948.10	942.8
Soybean meal	420.0	420.0	410.0	420.0	525.0	525.0	525.0	525.0
Soybean or corn oil	40.0	40.0	37.0	40.0	40.0	40.0	42.0	40.0
DDGS	150.0	150.0	150.0	150.0	200.0	200.0	200.0	200.0
PGF GMOS^e^	500.0	500.0	500.0	500.0	240.0	240.0	240.0	240.0
Lysine HCl	11.50	11.50	7.90	11.50	10.00	10.00	6.20	10.00
L-Threonine	4.50	4.50	2.80	4.50	3.70	3.70	1.80	3.70
DL-Methionine	2.10	2.10	0.40	2.10	2.40	2.40	0.70	2.40
Limestone					12.0	12.0	12.0	12.0
Monocalcium phosphate					5.10	5.10	5.10	5.10
PGF 3 lb VTM								
*L*-Tryptophan	1.40	1.40	0.70	1.40	0.70	0.70	0.10	0.70
TBCC								
Salt	8.00	8.00	8.00	8.00	8.00	8.00	8.00	8.00
PGF oat blend	200.0	200.0	200.0	200.0				
Mecadox	25 g/ton in each Phase 2 diet							
Peptiva		9.90	9.90	9.90		9.90	9.90	9.90
blended protease&MOS		1.10	1.10			1.10	1.10	
MOS				0.44				0.44
Total	2000.0	2000.0	2000.0	2000.0	2000.0	2000.0	2000.0	2000.0
Formulated nutrient content								
ME, kcal/kg	3288	3288	3288	3288	3262	3262	3262	3262
SID Lys, %	1.40	1.40	1.25	1.40	1.35	1.35	1.20	1.35
Ile:Lys	0.55	0.55	0.61	0.55	0.55	0.55	0.65	0.55
Thr:Lys	0.62	0.62	0.62	0.62	0.62	0.62	0.62	0.62
Trp:Lys	0.20	0.20	0.20	0.20	0.19	0.19	0.19	0.19
TSAA:Lys	0.58	0.58	0.58	0.58	0.58	0.58	0.58	0.58
Val:Lys	0.67	0.67	0.75	0.67	0.67	0.67	0.75	0.67
Ca, %	0.67	0.75	0.75	0.75	0.70	0.70	0.70	0.70
Available P, %	0.40	0.40	0.40	0.40	0.40	0.40	0.40	0.40
Lactose, %	11.0	11.0	11.0	11.0	4.0	4.0	4.0	4.0
Analyzed content								
Crude protein, %	21.3	21.2	19.9	21.3	21.6	22.3	21.2	21.4
Lys, %	1.51	1.47	1.35	1.54	1.47	1.48	1.28	1.52

^a^*Abbreviations*: *DDGS* Dried distillers grains with solubles, *PGF GMOS* Pipestone Grow-finish XX, *VTM* Vitamin/mineral trace mix, *TBCC* Tribasic copper chloride, *ME* Metabolizable energy, *SID* Standardized ileal digestible, *TSAA* Total sulfur amino acids

^b^PMP **=** PepM_Pro diet

^c^PMP90 = PepM_Pro(90) diet

^d^PM = PepM diet

^e^GF GMOS is a product from PIPESTONE (Pipestone, MN 56164, USA). It contains ingredients such as whey and specialty soy products, but it does not contain plasma

The experimental diets consisted of Control (Con; 274 pigs; formulated to meet nutrient requirement according to the NRC (2012) [47] guidelines; no supplementation with Peptiva, MOS or protease), Peptiva-MOS (PepM; 272 pigs; control diet supplemented with Peptiva and MOS), Peptiva-MOS with exogenous protease (PepM_Pro; 276 pigs; control diet supplemented with Peptiva, MOS and protease), and Peptiva-MOS with exogenous protease but with reduced amino acid content (PepM_Pro(90); 275 pigs dietary amino acid content at 90% of recommended NRC (2012) [47]; met recommended requirements for all other nutrients; supplemented with Peptiva, MOS and protease). Peptiva is a commercial product manufactured by Vitech Bio-Chem Corporation (Glendale, CA, USA) that consists of fish peptides, porcine digests and microbial peptides. MOS from *Saccharomyces cerevisiae* and exogenous protease from *Aspergillus Niger* were also products from the same company. More detailed information on the three commercial products used in this trial is provided in Additional file 2: Supplementary File 1. In all experimental diets, Peptiva-MOS was included at 0.3% as recommended by the manufacturer for use in commercial swine operations (Table 1).

### Growth performance and health assessment

Weaned pigs were assessed upon arrival to the facility. Pigs that were injured, sick, or too small were housed separately and not included in the trial. Pigs used in the study were randomly assigned to pens at weaning based on visual weight estimation. Treatments were randomized to pens within blocks (according to barn location) based on mean pen weight to achieve ≤ 10% covariance in pen weight between pens within treatment. Pens of pigs were weighed using a pen scale (accuracy of +/− 2.5 kg) at d 0 (barn entry), 14 (mid Phase II), 35 (end of Phase III), and 49 (2 weeks on a common diet after the end of Phase III). The swine facility was equipped with a single M-Series FEEDPro system (Feedlogic by ComDel Innovation, Willmar, MN 56201; accuracy of +/− 0.03%) for feeding that was used to monitor feed dispensed and disappearance for each pen. Diarrhea assessment was performed by pen from d 0 to d 10, the period of highest likely incidence, which overlapped with Phase I and the beginning of Phase II. Fecal scoring followed a 4-category scale: score of ‘1’ for feces that were firm and shaped, score of ‘2’ if feces were soft and shaped, score of ‘3’ if feces were loose, and a score of ‘4’ if feces were watery. Scores of ‘1’ and ‘2’ were considered healthy feces, while scores of ‘3’ and ‘4’ represented diarrhea. Each pen was observed by a single trained technician who assigned the relative proportion of visible feces within each category, as well as an overall pen score.

### Fecal sample collection

During the trial, two individuals from each pen fed the Con, PepM or PepM_Pro diets were randomly selected for collection of fecal samples at the end of Phase II and at the end of Phase III. Fecal samples were collected by rectal palpation, then stored frozen (− 20 °C). At the end of the animal trial, representative pens for each experimental diet were identified based on body weight, from which 10 pigs from the pool of available fecal samples were randomly selected for bacterial composition analysis.

### Isolation of microbial genomic DNA and sequencing of 16S rRNA gene amplicons

Microbial genomic DNA was isolated from fecal samples using the repeated bead beating plus column method, as previously described [48]. The V1-V3 region of the bacterial 16S rRNA gene was PCR-amplified using the 27F forward [49] and 519R reverse [50] primer pair. Generation of V1-V3 16S rRNA gene amplicons and Next Generation Sequencing using an Illumina MiSeq (2X300) platform were performed by the South Dakota State University Genomic Sequencing Facility.

### Computational analysis of PCR-generated 16S rRNA amplicon sequences

Unless specified, datasets were analyzed using custom written Perl scripts [51]. Overlapping raw forward and reverse reads from the same flow cell clusters were assembled into contigs using the ‘make.contigs’ command from MOTHUR (v 1.44) [52]. Eight of the 60 samples collected did not yield a sufficient number of 16S rRNA contigs, and these were not included in the analysis. The number of samples from each group that were used in the analysis was: Con PII (8); Con PIII (7); PepM PII (10); PepM PIII (9); PepM_Pro PII (8); PepM_Pro PIII (10).

Assembled 16S rRNA V1-V3 contig sequences were then screened to meet the following criteria: presence of both intact 27F (forward) and 519R (reverse) primer nucleotide sequences, length between 400 and 580 nt, and an average Phred quality score of at least Q33. Following quality screens, sequence reads were aligned, then clustered into Operational Taxonomic Units (OTUs) at a genetic distance cutoff of 4% sequence dissimilarity [53, 54]. A clustering cutoff of 3% is most commonly used for the 16S rRNA gene, but it was defined based on full-length sequences, so it is not necessarily suitable when analyzing sub-regions because nucleotide sequence variability is not the same throughout the entire 16S rRNA gene. If 3% is the standard clustering cutoff for V4 or V4-V5 regions, then a higher cutoff can be justified for the V1-V3 region, since it has much higher variability than other regions of the 16S rRNA gene [55]. OTUs were screened for chimeric sequences using the ‘chimera.uchime’ and ‘chimera.slayer’ commands from the MOTHUR open source software package [52]. Non-chimeric OTUs were then screened to assess the integrity of their 5′ and 3′ ends using an alignment-based approach. When compared to their closest match of equal or longer sequence length from the National Center for Biotechnology Information (NCBI) ‘nt’ database, as determined by BLAST (Basic Local Alignment Search Tool) [56], OTUs with more than five nucleotides missing from the 5′ or 3′ end of their respective alignments were discarded as artifacts. For OTUs with only one or two assigned reads, only sequences that had a perfect or near perfect match to a sequence in the NCBI ‘nt’ database were kept for analysis (the alignment had to span the entire sequence of the OTU, and a maximum of 1% of dissimilar nucleotides was tolerated).

After removal of sequence chimeras and artifacts, RDP Classifier [57] and BLAST [56] were used for taxonomic assignment of filtered OTUs. Additional information on valid species belonging to taxa of interest was obtained from the List of Prokaryotic Names with Standing in Nomenclature (LPSN - http://www.bacterio.net) [58].

### Statistical analyses

Growth performance was analyzed using the PROC MIXED procedure of SAS (Version 9.4; SAS Inst. Inc., Cary, NC), with pen as the experimental unit and pen as the random variable. The contrast statement was used for pre-planned comparisons. Chi-squared analysis was used to evaluate fecal scores. Differences between treatment means were tested using Tukey’s adjusted means test when a significant interaction was observed. Means were considered to be significantly different when *P* ≤ 0.05, and a tendency towards statistical significance was indicated when 0.05 < *P* ≤ 0.10.

Using R (Version R-3.2.3), ANOVA (command aov) and post hoc Tukey Honest Significant Difference (command TukeyHSD) analyses were performed to compare alpha diversity indices. The Kruskal-Wallis sum-rank test was used (command ‘kruskal.test’) to determine if the abundances of selected taxa varied across sample groups. The pairwise Wilcoxon sum-rank test (command ‘pairwise.wilcox.test’) was used to compare abundances between sample group pairs, with the Benjamini-Hochberg correction for controlling false discovery rate. Statistical significance was set at *P* ≤ 0.05.

### Next generation sequencing data accessibility

Raw sequence data are available from the NCBI Sequence Read Archive under Bioproject PRJNA769941.

## Results

### Effects of experimental diets on the production performance of nursery pigs

The potential effects of Peptiva-MOS with or without the addition of exogenous protease were evaluated in weaned pigs fed a standard phase nursery diet. No significant difference in fecal scores was found amongst groups of pens that were assigned to different dietary treatments; the average pen fecal score for each group was 3 or greater over the 10-day observation period. No significant effect of experimental diets on body weight was observed during the trial (Table 2). However, pigs fed the PepM_Pro diet showed higher average daily gain between d 15 and d 35 compared to control (*P* < 0.05). Higher gain:feed was observed for PepM pigs compared to Con when experimental diets were fed (d 15 to d 35 and d 0 to d 35), but lower Gain:Feed was observed for the PepM and PepM_Pro diets compared to Con during the period when pigs from all groups were fed a common diet (d 36–49).

**Table 2:** Growth performance of weaned pigs under four different dietary treatments

	Con	PepM^1^	PepM_Pro^2^	PepM_Pro(90)^3^	SEM	*P*-value
BW, kg						
d 0	6.9	6.8	6.9	7.0	0.11	0.453
d 14	12.4	12.4	12.4	12.4	0.18	0.791
d 35	23.8	24.2	24.4	23.7	0.23	0.139
d 49	35.1	35.0	35.4	34.6	0.27	0.196
ADG, kg/d						
d 0-14	0.355	0.359	0.357	0.360	0.012	0.985
d 15-35	0.534^b^	0.558^a,b^	0.568^a^	0.536^b^	0.010	0.017
d 36-49	0.851	0.829	0.833	0.823	0.017	0.404
d 0-35	0.443	0.458	0.461	0.447	0.007	0.204
d 0-49	0.563	0.569	0.574	0.558	0.006	0.259
ADF, kg/d						
d 0-14	0.349	0.342	0.371	0.383	0.013	0.133
d 15-35	0.875	0.809	0.842	0.817	0.028	0.296
d 36-49	1.303^a^	1.429^b^	1.419^a,b^	1.437^b^	0.044	0.050
d 0-35	0.611	0.575	0.606	0.600	0.016	0.364
d 0-49	0.847	0.863	0.882	0.883	0.011	0.089
g:f, kg:kg						
d 0-14	1.009^x,y^	1.100^x^	0.962^x,y^	0.921^y^	0.050	0.082
d 15-35	0.603^a^	0.707^b^	0.674^a,b^	0.657^a,b^	0.028	0.028
d 36-49	0.652^a^	0.583^b^	0.591^b^	0.579^b^	0.016	0.007
d 0-35	0.726^a^	0.819^b^	0.765^a,b^	0.745^a,b^	0.020	0.009
d 0-49	0.666^a^	0.662^a^	0.653^a,b^	0.633^b^	0.007	0.007

Experimental diets (Phase II and Phase III) were fed for 35 d (indicated as d 0–35, corresponding to d 7 to d 42 post-weaning), followed by a common diet for 14 d (indicated as d 36–49)

Means with different superscripts within a row were found to be different at a significance threshold of *P* ≤ 0.05 (^a,b^) or to show a tendency when at 0.05 < *P* < 0.10 (x,y), based on the Tukey honest significant difference test

^1^Control diet supplemented with Peptiva and MOS (formulated to provide 100% of required amino acids for weaned pigs – NRC 2012)

^2^Control diet supplemented with Peptiva, MOS and a protease (formulated to provide 100% of required amino acids for weaned pigs – NRC 2012)

^3^Control diet supplemented with Peptiva, MOS and a protease (formulated to provide 90% of required amino acids for weaned pigs – NRC 2012)

The potential of supplementation with Peptiva, MOS and exogenous protease to compensate for reduced inclusion of amino acids in nursery diets was investigated in weaned pigs fed a diet providing only 90% of the recommended NRC (2012) [47] guidelines for amino acid requirement. No significant difference in body weight, average daily gain, or feed intake between the PepM_Pro(90) group and Con was observed during the trial. While no difference in Gain:Feed was observed between PepM_Pro(90) pigs and Con pigs when experimental diets were fed, the former showed lower Gain:Feed compared to the latter (*P* < 0.05) when pigs from all groups were fed a common diet (d 36–49).

### Taxonomic profile of fecal bacterial communities

The potential effects of Peptiva-MOS supplementation in the presence or absence of exogenous protease on the gut microbial profiles of nursery pigs were investigated using fecal bacterial communities as a proxy. Streptococcaceae (Firmicutes) and Bacteroidaceae (Bacteroidetes) were the only taxa found to be different amongst treatments at the same time point, with both families in lower abundance in samples from Con-Phase III pigs compared to samples from PepM_Pro pigs at Phase III (*P* < 0.05; Table 3).

Most differences in taxonomic profiles were observed between Phase II and Phase III samples for matching treatment pairs. Actinobacteria, for instance, were found in higher abundance (*P* < 0.05) in Phase II samples (means ranging between 2.26% and 3.03%) compared to Phase III samples (means ranging between 0.69% and 1.13%). While Firmicutes were maintained within a narrow range across groups (78.55–87.56%), Lactobacillaceae (3.14–8.90% vs 15.40–21.64%), Peptostreptococcaceae (0.20–0.95% vs 1.82–2.71%) and Streptococcaceae (0.35–2.05% vs 5.15–19.60%), three families affiliated with this phylum, were found in higher abundance in Phase III samples compared to Phase II samples for all dietary treatments (*P* < 0.05; Table 3).

### Comparative analysis of fecal bacterial composition by alpha and beta diversity

**Fig. 1:**
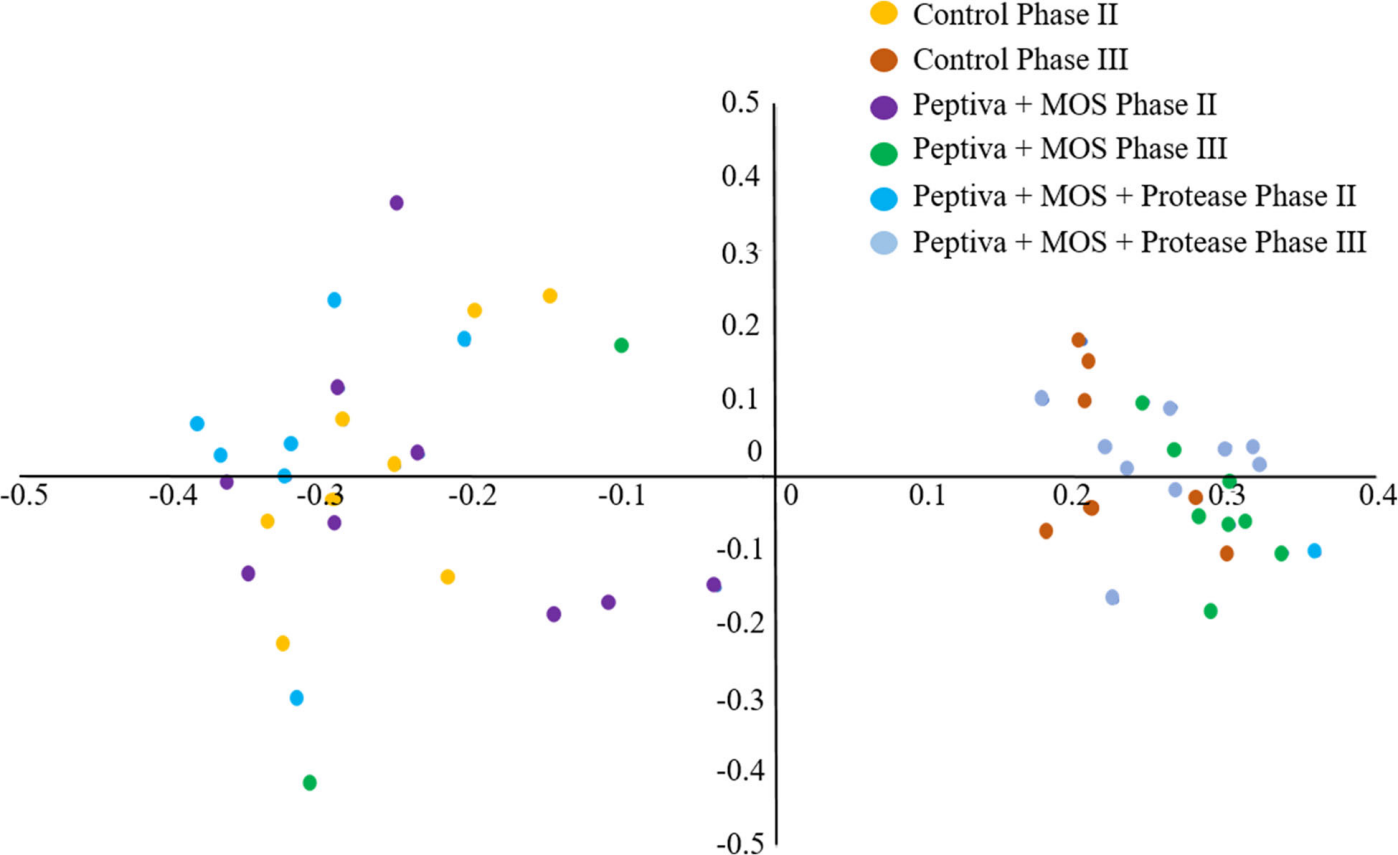
Comparison of fecal bacterial communities amongst the different treatment groups for Phase II and Phase III. Principal Coordinate Analysis (PCoA) was performed using a Bray-Curtis distance matrix. The x and y axes correspond to Principal Components 1 (PCo1) and 2 (PCo2)

To gain further insight, an analysis based on OTU composition was performed, resulting in the identification of 4332 OTUs across all samples. No significant differences were found for the alpha diversity indices tested (*P* > 0.05; Table 4). Clustering of samples by PCoA was consistent with the taxonomic profiles described above, with two distinct groups observed: one consisting of 24 of the 25 Phase II samples while the other grouped 25 of the 27 Phase III samples (Fig. 1).

**Table 3:** Average relative abundance of main taxonomic groups in representative fecal samples from three dietary treatments at Phase II and Phase III, %

OTUs	ConII^**1**^	ConIII^**2**^	PmII^**3**^	PmIII^**4**^	PmPII^**5**^	PmPIII^**6**^
Actinobacteria^#^	2.26^ac^	0.69^b^	3.03^a^	1.13^bc^	2.69^a^	0.83^b^
Bacteroidetes	12.95	8.59	10.54	7.27	14.25	6.83
Bacteroidaceae^#^	1.31^a^	0.05^b^	0.45^a^	0.85^abc^	0.54^ac^	0.10^c^
Porphyromonadaceae	1.16	4.38	2.21	0.28	1.31	0.38
Prevotellaceae	9.20	5.70	5.79	5.31	9.89	5.43
Other Bacteroidetes^a^	1.28	2.41	2.10	0.83	2.52	0.93
Firmicutes	79.39	83.67	80.65	87.56	78.55	87.71
Acidaminococcaceae^#^	3.38^ab^	0.14^b^	1.59^a^	0.32^ab^	1.36^a^	0.19^b^
Clostridiaceae 1	2.41	4.70	1.80	4.38	2.50	2.05
Cl Inc Sedis_XIII^#^	2.36^abc^	0.96^b^	3.65^a^	1.31^bc^	4.28^ac^	1.03^b^
Erysipelotrichacea^#^	15.47^abc^	7.49^b^	19.66^c^	10.27^ab^	22.11^ac^	7.18^b^
Eubacteriaceae^#^	1.44^ad^	0.17^cd^	0.75^ad^	0.19^bcd^	0.56^d^	0.11^bc^
Lachnospiraceae^#^	19.64	9.18	16.48	13.16	10.52	13.82
Lactobacillaceae^#^	3.14^a^	21.64^bc^	8.90^ac^	21.22^b^	4.41^a^	15.40^bc^
Peptostreptococcaceae^#^	0.20^a^	2.68^b^	0.28^a^	2.71^b^	0.95^a^	1.82^b^
Ruminococcaceae	21.16	17.56	16.40	14.23	19.67	15.27
Streptococcaceae^#^	0.35^a^	5.15^b^	2.05^a^	10.32^bc^	1.39^a^	19.60^c^
Other Firmicutes^&^	9.84	13.99	9.09	9.47	10.81	11.24
Proteobacteria^#^	0.73^ab^	0.77^ab^	1.11^a^	0.37^ab^	0.45^ab^	0.17^b^
Spirochaetes	2.06	0.55	2.02	0.56	1.40	0.29
Other phyla^$^	0.69	0.59	0.43	0.45	0.66	0.80
Unclassified bacteria^$^	1.92	5.14	2.22	2.65	1.99	3.36

Different superscripts in the same row indicate that taxa were significantly different by the Wilcoxon pairwise test (*P* < 0.05)

^1^Control Phase II; ^2^Control Phase III; ^3^Peptiva + MOS Phase II; ^4^Peptiva + MOS Phase III; ^5^Peptiva + MOS + protease Phase II; ^6^Peptiva + MOS + protease Phase III

^#^ Taxa showing a statistically significant difference (*P* < 0.05) across all 6 groups based on the Kruskal-Wallis rank sum test

^a^Statistical test not performed due to heterogeneity of taxonomic group

### Composition analysis by OTU profile

Taxonomic profiles had indicated that the effects of Peptiva-MOS with or without protease supplementation on fecal bacterial communities were more subtle than the impact of the transition between Phase II and Phase III diet formulations. However, these effects were not detected by PCoA. To explore potential differences at the level of individual bacterial species, a more in depth analysis focused on the most abundant OTUs, defined as OTUs with a mean relative abundance of at least 1% in at least one set of samples, was performed.

Of the 32 most abundant OTUs identified in this study, three were found at significantly different levels between Con samples and samples from a Peptiva-MOS treatment during the same phase (*P* < 0.05; Table 5). Amongst Phase III treatment groups, Ssd-00039 showed significantly higher representation in the samples from the PepM_Pro treatment compared to Con samples (*P* < 0.05). Ssd-00928 showed an opposite composition profile, with higher abundance in Con samples from Phase II compared to samples from the PepM_Pro treatment during Phase II. For both of these OTUs, the abundance in PepM samples appeared to be intermediate between Control and PepM_Pro. Ssd-01079 showed a very distinct composition pattern, with samples from PepM at Phase III being lower than Con and PepM_Pro samples at Phase III.

**Table 4:** Alpha diversity indices and coverage from three dietary treatments at Phase II and Phase III. Values are presented as means

Index	ConII^**1**^	ConIII^**2**^	PmII^**3**^	PmIII^**4**^	PmPII^**5**^	PmPIII^**6**^
OTUs^#^	364	509	401	447	438	464
Ace	638	849	622	659	658	692
Chao1	546	753	716	693	757	785
Shannon	4.13	4.66	4.32	4.38	4.39	4.37
Simpson	0.054	0.034	0.043	0.046	0.041	0.058
Coverage, %	97.2	96.0	96.8	96.5	96.5	96.4

^#^ Alpha index showing a statistically significant difference (*P* < 0.05) across all six groups based on the Kruskal-Wallis rank sum test

No statistically significant differences by pairwise comparisons

^1^Control Phase II; ^2^Control Phase III; ^3^Peptiva + MOS Phase II; ^4^Peptiva + MOS Phase III; ^5^Peptiva + MOS + protease Phase II; ^6^Peptiva + MOS + protease Phase III

**Table 5:** Mean relative abundance of OTUs showing a significant difference in representative fecal samples from three dietary treatments at Phase II and Phase III, %

OTUs	ConII^**1**^	ConIII^**2**^	PmII^**3**^	PmIII^**4**^	PmPII^**5**^	PmPIII^**6**^	Closest taxon (id.%)
Ssd-00001^#^	0.33^a^	10.57^b^	2.03^a^	11.02^b^	1.92^a^	6.06^b^	*L. amylovorus* (99%)
Ssd-00002^#^	0.03^a^	2.86^b^	0.12^a^	1.40^b^	0.08^a^	2.61^b^	*L. johnsonii* (99%)
Ssd-00014^#^	0.07^a^	2.24^b^	0.20^a^	2.26^b^	0.70^a^	1.45^b^	*T. glycolicus* (97%)
Ssd-00019^#^	2.11	5.43	5.25	5.73	1.48	3.97	*L. reuteri* (99%)
Ssd-00021^#^	1.04^ab^	0.10^a^	0.75^b^	0.52^a^	1.33^ab^	0.05^a^	*P. copri* (95%)
Ssd-00039^#^	0.27^a^	4.46^b^	1.72 ^a^	8.95^bc^	1.12 ^a^	17.19^c^	*St. alactolyticus* (99%)
Ssd-00123^#^	7.23^abc^	0.11^a^	7.45^b^	2.83^ac^	9.32^bc^	0.23^ac^	*L. vitulina* (87%)
Ssd-00134^#^	1.61^ab^	3.06^b^	1.10^a^	2.57^b^	1.60^ab^	1.13^b^	*Cl. saccharoper.* (97%)
Ssd-00188^#^	0.13^a^	0.57^ab^	0.77^ab^	1.27^ab^	0.04^a^	2.25^b^	*E. rectale* (99%)
Ssd-00304^#^	0.22^a^	0.88^ab^	0.49^ab^	1.42^ab^	0.49^ab^	0.95^b^	*A. senegalensis* (84%)
Ssd-00308^#^	1.49^ab^	0.55^a^	4.35^b^	1.41^ab^	4.12^b^	0.98^a^	*H. biformis* (97%)
Ssd-00416^#^	2.67^abc^	0.06^a^	1.28^b^	0.08^ac^	0.82^bc^	0.08^a^	*Ph. succinatutens* (95%)
Ssd-00706^#^	0.48^ab^	1.31^a^	0.29^b^	1.05^ab^	0.56^ab^	1.05^a^	*L. paracasei* (81%)
Ssd-00840^#^	1.20^ab^	0.12^a^	1.69^b^	0.47^a^	1.83^b^	0.23^a^	*Co. aerofaciens* (98%)
Ssd-00892^#^	0.71^ab^	0.42^a^	2.30^b^	0.44^a^	1.66^ab^	0.35^a^	*So. moorei* (89%)
Ssd-00928^#^	1.14^a^	0.12^bc^	0.60^ab^	0.07^c^	0.36^bc^	0.08^c^	*R. gnavus* (96%)
Ssd-00993^#^	1.33^ab^	0.33^ab^	1.61^a^	0.25^b^	1.67^ab^	0.20^b^	*F. cylindroides* (88%)
Ssd-01079^#^	0.62^ab^	0.78^a^	1.72^a^	0.04^b^	1.25^a^	0.27^a^	*M. australiensis* (84%)
Ssd-01080^#^	0.31	0.19	0.53	0.13	1.59	0.21	*I. massiliensis* (92%)
Ssd-01081^#^	1.34	0.60	0.11	0.36	0.46	0.75	*B. pachnodae* (81%)
Ssd-01244^#^	2.05^a^	<0.01^b^	0.26^a^	0.00^b^	0.77^a^	0.00^b^	*R. bromii* (92%)
Ssd-01246^#^	0.64^a^	<0.00^b^	0.49^a^	<0.01^b^	1.22^a^	<0.01^b^	*Sh. azabuensis* (97%)

^**#**^ *P* < 0.05 based on the Kruskal-Wallis rank-sum test

Different superscripts in the same row indicate that OTUs were significantly different by the Wilcoxon pairwise test (*P* < 0.05)

^1^Control Phase II; ^2^Control Phase III; ^3^Peptiva + MOS Phase II; ^4^Peptiva + MOS Phase III; ^5^Peptiva + MOS + protease Phase II; ^6^Peptiva + MOS + protease Phase III

*Abbreviations*: *A* Anaeromassilibacillus, *B* Blautia, *Cl* Clostridium, *Co* Collinsella, *E* Eubacterium, *F* Faecalibacterium, *H* Holdemanella, *I* Ihubacter, *L* Lactobacillus, *M* Mahella, *P* Prevotella, *Ph* Phascolarctobacterium, *R* Ruminococcus, *saccharoper* saccharoperbutylacetonicum, *Sh* Sharpea, *So* Solobacterium, *St* Streptococcus, *T* Terrisporobacter

Sixteen of the 32 most abundant OTUs were found to vary between Phase II and Phase III when comparing samples from groups receiving the same treatment, i.e. either Con pairs, PepM pairs and/or PepM_Pro pairs. Six of these 16 OTUs were found at significantly different abundance levels when comparing samples from all matching treatment pairs between Phase II and Phase III diets. Ssd-00001, Ssd-00002, Ssd-00014, and Ssd-00039 were lower in Phase II samples compared to Phase III samples, while Ssd-01244 and Ssd-01246 displayed an opposite profile by being higher in Phase II samples compared to Phase III samples. The remaining ten OTUs displayed a difference in abundance between Phase II and Phase III for some of the treatment pairs. Ssd-00928 was in higher abundance in Phase II compared to Phase III from Con and PepM treatments pairs, while Ssd-00416 and Ssd-00840 were more highly represented in Phase II samples for the PepM and PepM_Pro treatment pairs. Differences in abundance for OTUs Ssd-00123, Ssd-00134, Ssd-00892, Ssd-00993 and Ssd-01079 between Phase II and Phase III were observed only for the PepM treatment pair, while differences for Ssd-00188 and Ssd-00308 were detected only for the PepM_Pro treatment pair.

## Discussion

Based on available information, the three feed supplements tested in this study were hypothesized to work through separate mechanisms. Dietary peptides have previously been reported to benefit animal performance by increasing the availability of short peptides and free amino acids for absorption in comparison to intact proteins [59]. Indeed, the transport of amino acids in the form of peptides has been previously demonstrated to be a faster route of uptake compared to free amino acids [60, 61]; peptides with two or three amino acids can be transported into a cell by the PepT1 transporter for the same energy expenditure required to transport a single free amino acid [62, 63]. In addition to providing amino acids, certain peptides, referred to as bioactive, can perform other functions. A number of studies have for instance observed improved average daily gain, average daily feed intake, digestibility, and feed efficiency as a result of dietary supplementation of nursery diets with different types of antimicrobial peptides, such as lactoferrin, cecropin, defensin, or plectasin [39, 40, 42, 44, 45]. In contrast, MOS are branched molecules made of glucose, mannose and N-acetylglucosamine [64] that can impact gut microbiomes through different mechanisms. They can act as high affinity ligands for binding to pathogens, thus minimizing the risk of pathogen attachment to gut epithelial cells and preventing the onset of enteric infections [65, 66]. MOS can also function as prebiotics, i.e. by providing substrates that can be metabolized by beneficial symbionts of gut microbiomes. Reports on the impact of MOS inclusion in nursery pig diets have so far been inconsistent, ranging from no obvious benefits according to certain studies [28, 67] to improved growth and feed efficiency in others [30, 68, 69]. Interestingly, dietary supplementation of sow diets with MOS in the last 2–3 weeks of gestation and during lactation has been reported to improve piglet growth rate [70]. The addition of exogenous proteases in nursery diets has been reported to increase growth performance, protein digestibility, nutrient transport efficiency, as well as apparent ileal digestibility [23, 24, 27].

To gain further insight on these feed additives or supplements, the combination of a peptide product (Peptiva) with MOS and an exogenous protease was tested for potential benefits to the performance of nursery pigs. Dietary inclusion of Peptiva and MOS was found to be beneficial to nursery pigs, resulting in higher gain:feed, an indicator of feed efficiency. Under the conditions of this study, the addition of an exogenous protease to supplementation with Peptiva and MOS increased average daily gain, but the benefits of this combination to feed efficiency (gain:feed) were not as clear compared to inclusion of Peptiva and MOS without protease supplementation. Regardless, the benefits of Peptiva-MOS dietary inclusion during the nursery stage were not maintained after pigs were transitioned to a non-supplemented diet. While there was no statistical difference in ADG amongst experimental groups and the control during the non-supplemented period (d 36-49), the former showed significantly higher intake, resulting in lower gain:feed. One possible explanation for these observations could be that the presence of feed additives in the gut environment promoted favorable physiological conditions for efficient use of feed, such as increased digestion and/or host absorption. This could possibly have been mediated through changes in gut microbial community composition as observed in this study or by modulation of host cell activities. As the animal performance results suggest that these functionalities required a constant input of the feed additives in order to be sustained, pigs from the experimental groups would have required higher intake of feed in order to maintain their ADG when supplementation was withdrawn.

We have previously reported that Peptiva can affect the composition of fecal bacterial communities in weaned pigs [51]. Considering that this peptide product was used in combination with a prebiotic (MOS) in this current report, we looked for differences in bacterial composition of fecal samples that could be indicative of potential effects on gut microbiome profiles. Three candidate bacterial species (OTUs) were found to have significantly different abundances between control samples and treatment samples during the same phase. Ssd-000928 and Ssd-01079 likely corresponded to novel bacterial species, since their respective 16S rRNA gene sequences only showed limited identity to their closest valid relative. In contrast, SD_Ssd-00039 was found to have 99% sequence identity to *S. alactolyticus*, and thus may have corresponded to a strain of this species. As this OTU represented 86.6–87.7% of Streptococcaceae sequences in Phase III samples across the different treatments, the change in its abundance was likely why Streptococcaceae were also found to vary across the different treatments. *S. alactolyticus* was originally isolated from the swine intestinal tract and from chicken feces [71], and it was later reported to be a predominant commensal in the swine colonic environment [72, 73]. *S. alactolyticus* is a lactic acid producing bacterial species with several reported beneficial effects for its hosts [74], such as suppressing the growth of intestinal pathogens [75, 76] and enhancing immune functions [77, 78]. In Phase III samples, the average abundance of SD_Ssd-00039 in samples from PepM_Pro supplemented pigs was 3.9 times higher than in Controls. Notably, Ssd-00001 was the most abundant OTU in Phase III samples from Controls and PepM supplemented pigs. As Ssd-00001 is likely a strain of *L. amylovorous* and also predicted to be a lactate producer [79], it would be of great interest to compare the metabolic capabilities and properties of Ssd-00001 and Ssd-00039 in order to determine the potential impact of this change in bacterial composition on the gut environment of nursery pigs.

Considering the importance of the gut microbiome for the health and nutrition of their host, the establishment of stable microbial communities in the gut of young animals is critical for their health. While bacterial succession can occur throughout the lifetime of a pig as a result of events such as diet change and stress [80], weaning represents one of the most disruptive events for gut microbial composition. Indeed, not only is the transition from milk to solid feed dramatically altering the range of substrates available for gut symbionts to metabolize, it is also acting on a microbial environment with limited resistance and resilience. Until the gastrointestinal tract of weaned pigs has adjusted to digesting unfamiliar substrates such as starch, these remain available for gut symbionts to utilize with limited competition from the host. In intensive swine production systems, weaned pigs are commonly fed phase diets that start with high quality and easily digestible ingredients to increase palatability as well as nutrient accessibility for the immature gut. Gut adaptation to solid feed allows for replacement of high quality ingredients with alternative feedstuffs during the different phases of the nursery stage, resulting in reduced ingredient costs to offset the increase in feed intake. While necessary from a nutrition and management standpoint, phase diets may contribute to microbiome instability in young pigs. However, a deeper understanding of the beneficial gut symbionts that need to be stably established and of their metabolic capabilities would allow to fine tune phase diets to minimize dramatic changes in microbial composition during diet transitions. This would not only contribute to maintain the health and growth performance of pigs at weaning, but also throughout their productive life [81, 82].

Major differences in fecal bacterial composition were observed between Phase II and Phase III that were independent of treatments. These variations in profile were consistent with microbial succession taking place in the gut of nursery pigs between the two phases, and are consistent with previously published reports [51, 83, 84]. Among the major composition changes observed, members of the phylum Actinobacteria were found in higher abundance in Phase II compared to Phase III. Of the 32 most abundant OTUs that were analyzed individually, only Ssd-00840 was found to be affiliated to Actinobacteria. In Phase II samples, it represented 53.1 to 68.0% of sequence reads per group that were affiliated to Actinobacteria.

Three families affiliated to Firmicutes (Streptococcaceae, Lactobacillaceae and Peptostreptococcaceae) were found to be significantly more abundant in Phase III samples compared to Phase II samples when matching treatment pairs were compared. In contrast, other well-represented Firmicutes families such as Lachnospiraceae and Ruminococcaceae were not found to vary across treatments or time points. As discussed in a previous section, Ssd-00039 was the main OTU affiliated to Streptococcacea, and it would be predicted to function as a lactate producer. Similarly, two main OTUs affiliated to Lactobacillaceae (Ssd-00001 and Ssd-00002) that were also found to be statistically different between Phase II and Phase III samples from the same treatment were also predicted to be lactate producers. *Lactobacilli* are typically abundant in swine gut bacterial communities, and they play important roles in maintaining the health status of the host gastro-intestinal tract [85–88]. Ssd-00001 was likely a strain of *L. amylovorus*, which has been reported to express antimicrobial activity against enteric pathogens and to produce large quantities of lactic acid [79, 89]. *L. amylovorus* express surface (S)-layer proteins that provide strong adhesive properties for interactions with enterocytes and the extracellular matrix of the host [90]. S-layer proteins have been reported to act as antigen delivery vehicles for host cells [91], thus aiding in activating the innate immune system and contributing to gut health [92]. Ssd-00002 was likely a strain of *L. johnsonii*, a species associated with antimicrobial effects within the gut environment of post-weaned pig [93]. *L. johnsonii* has been shown to have probiotic qualities and to express aggregation-promoting factor proteins, which, based on their structure and location, may provide similar functions to S-layer proteins [94]. Ssd-00014 was the main abundant OTU affiliated to Peptostreptococcaceae, and its closest valid relative was *Terrisporobacter mayombei*. First described as *Clostridium mayombei*, this bacterial species was isolated from a soil-feeding termite [95], and it has been characterized as an acetogen, using H_2_ and CO_2_ as substrates, and found to be capable of fermenting monosaccharides (glucose, fructose, xylose), as well as amino acids such as alanine, glutamate, serine and valine [96].

## Conclusion

Together, the results from this study support that the combination of Peptiva, MOS and protease can benefit the performance of weaned pigs during the nursery phase, and that these feed additives can modulate the composition of the swine gut microbiome during this period. Three candidate bacterial species identified as OTUs were found to differ in abundance in supplemented animals compared to controls during the same phase. Notably, as one of these OTUs, Ssd-00039, was the most abundant candidate bacterial species identified in this study, it would be of great interest to determine its metabolic capabilities in order to determine the potential impact of this change in bacterial composition on the gut environment of nursery pigs.

## Supplementary Information

**Additional file 1: Supplementary Table 1.** Relative abundance of all curated OTUs that were identified in fecal samples from three dietary treatments at Phase II and Phase III.

**Additional file 2: Supplementary file 1.** Product description for Peptiva, MOS and exogenous protease from the manufacturer (Vitech Bio-Chem Corporation (Glendale, CA, USA).

## Data Availability

The raw sequencing reads of this study have been deposited in the NCBI Sequence Read Archive (SRA) database under BioProject PRJNA769941.

## Abbreviations

ANOVA: Analysis of variance
BLAST: Basic Local Alignment Search Tool
MOS: Mannose oligosaccharides
NCBI: National Center for Biotechnology Information
NRC: National Research Council
OTU: Operational taxonomic unit
PCoA: Principal Coordinate Analysis
RDP: Ribosomal Database Project
SCFA: Short-chain fatty acid
SDSU: South Dakota State University

## References

[1] de Lange CFM, Pluske J, Gong J, Nyachoti NM. Strategic use of feed ingredients and feed additives to stimulate gut health and development in young pigs. Livest Sci. 2010;134:124–34. doi: 10.1016/j.livsci.2010.06.117.

[2] Campbell JM, Crenshaw JD, Polo J. The biological stress of early weaned piglets. J Anim Sci Biotechnol. 2013;4:19. 10.1186/2049-1891-4-19. 10.1186/2049-1891-4-19 PMC3651348 23631414

[3] Pluske JR (2016). Invited review: aspects of gastrointestinal tract growth and maturation in the pre- and postweaning period of pigs. J Ani Sci..

[4] van Beers-Schreurs HMG, Nabuurs MJA, Vellenga L, Kalsbeek-van der Valk HJ, Wensing T, Breukink HJ. Weaning and the weanling diet influence the villous height and crypt depth in the small intestine of pigs and alter the concentrations of short-chain fatty acids in the large intestine and blood. J Nutr. 1998;128(6):947–53. 10.1093/jn/128.6.947. 10.1093/jn/128.6.947 9614152

[5] Dong GZ, Pluske JR (2007). The low feed intake in newly-weaned pigs: problems and possible solutions. Asian-Australas J Anim Sci..

[6] Pluske JR, Hampson DJ, Williams IH. Factors influencing the structure and function of the small intestine in the weaned pig: a review. Livest Prod Sci. 1997;51:215–36. doi: 10.1016/S0301-6226(97)00057-2.

[7] Hedemann MS, Jensen BB (2004). Variations in enzyme activity in stomach and pancreatic tissue and digesta in piglets around weaning. Arch Anim Nutr.

[8] Lallès J-P, Boudry G, Favier C, Le Floc’h N, Luron I, Montagne L (2004). Gut function and dysfunction in young pigs: physiology. Anim Res.

[9] Boudry G, Péron V, Le Huërou-Luron I, Lallès JP, Sève B (2004). Weaning induces both transient and long-lasting modifications of absorptive, secretory, and barrier properties of piglet intestine. J Nutr.

[10] Madec F, Bridoux N, Bounaix S, Jestin A (1998). Measurement of digestive disorders in the piglet at weaning and related risk factors. Prev Vet Med.

[11] Htoo JK, Araiza BA, Sauer WC, Rademacher M, Zhang Y, Cervantes M, Zijlstra RT (2007). Effect of dietary protein content on ileal amino acid digestibility, growth performance, and formation of microbial metabolites in ileal and cecal digesta of early-weaned pigs. J Anim Sci.

[12] Tactacan GB, Cho S-Y, Cho JH, Kim IH (2016). Performance responses, nutrient digestibility, blood characteristics, and measures of gastrointestinal health in weanling pigs fed protease enzyme. Asian-Australas J Anim Sci.

[13] Gresse R, Chaucheyras-Durand F, Fleury MA, Van de Wiele T, Forano E, Blanquet-Diot S (2017). Gut microbiota dysbiosis in postweaning piglets: understanding the keys to health. Trends Microbiol.

[14] Porter P, Kenworthy R. A study of intestinal and urinary amines in pigs in relation to weaning. Vet Sci Res J. 1969;10:440–7. doi: 10.1016/S0034-5288(18)34410-2. 5358407

[15] Dong G, Zhou A, Yang F, Chen K, Wang K, Dao D (1996). Effect of dietary protein levels on the bacterial breakdown of protein in the large intestine, and diarrhoea in early weaned piglets. Acta Vet Zootec.

[16] Gaskins HR. Intestinal bacteria and their influence on swine growth. In: Lewis AJ, Southern LL, editors. Swine nutrition, 2nd edition. Boca Raton: CRC Press; 2000. p. 585-608. 10.1201/9781420041842.

[17] Nahm KH (2003). Evaluation of the nitrogen content in poultry manure. Poult Sci J..

[18] Friedman M, Brandon DL (2001). Nutritional and health benefits of soy proteins. J Agric Food Chem.

[19] Jo JK, Ingale SL, Kim JS, Kim YW, Kim KH, Lohakare JD, Lee JH, Chae BJ (2012). Effects of exogenous enzyme supplementation to corn- and soybean meal-based or complex diets on growth performance, nutrient digestibility, and blood metabolites in growing pigs. J Ani Sci..

[20] de Souza ALP, Lindemann MD, Cromwell GL. Supplementation of dietary enzymes has varying effects on apparent protein and amino acid digestibility in reproducing sows. Livest Sci. 2007;109:122–4. doi: 10.1016/j.livsci.2007.01.113.

[21] Cowieson AJ, Ravindran V (2008). Effect of exogenous enzymes in maize-based diets varying in nutrient density for young broilers: growth performance and digestibility of energy, minerals and amino acids. Br Poult Sci.

[22] Yoon SY, Yang YX, Shinde PL, Choi JY, Kim JS, Kim YW, Yun K, Jo JK, Lee JH, Ohh SJ, Kwon IK, Chae BJ (2010). Effects of mannanase and distillers dried grain with solubles on growth performance, nutrient digestibility, and carcass characteristics of grower-finisher pigs. J Ani Sci..

[23] Rooke JA, Slessor M, Fraser H, Thomson RJ. Growth performance and gut function of piglets weaned at four weeks of age and fed protease-treated soya-bean meal. Anim Feed Sci Technol. 1998;70:175–90. doi: 10.1016/S0377-8401(97)00083-7.

[24] Guggenbuhl P, Wache Y, Wilson JW (2012). Effects of dietary supplementation with a protease on the apparent ileal digestibility of the weaned piglet. J Anim Sci.

[25] Wang H, Guo Y, Shih JCH. Effects of dietary supplementation of keratinase on growth performance, nitrogen retention and intestinal morphology of broiler chickens fed diets with soybean and cottonseed meals. Anim Feed Sci Technol. 2008;140:376–84. doi: 10.1016/j.anifeedsci.2007.04.003.

[26] Caine WR, Sauer WC, Tamminga S, Verstegen MWA, Schulze H (1997). Apparent ileal digestibilities of amino acids in newly weaned pigs fed diets with protease-treated soybean meal. J Ani Sci..

[27] Zuo J, Ling B, Long L, Li T, Lahaye L, Yang C, et al. Effect of dietary supplementation with protease on growth performance, nutrient digestibility, intestinal morphology, digestive enzymes and gene expression of weaned piglets. Anim Nutr. 2015;1:276–82. doi: 10.1016/j.aninu.2015.10.003. 10.1016/j.aninu.2015.10.003 PMC5940980 29767006

[28] White LA, Newman MC, Cromwell GL, Lindemann MD (2002). Brewers dried yeast as a source of mannan oligosaccharides for weanling pigs. J Anim Sci.

[29] Harper AF, Estienne MJ (2002). Efficacy of three potential alternatives to antimicrobial feed additives for weanling pigs. Prof Anim Sci.

[30] Davis ME, Maxwell CV, Brown DC, de Rodas BZ, Johnson ZB, Kegley EB, Hellwig DH, Dvorak RA (2002). Effect of dietary mannan oligosaccharides and (or) pharmacological additions of copper sulfate on growth performance and immunocompetence of weanling and growing/finishing pigs. J Anim Sci.

[31] Rozeboom DW, Shaw DT, Tempelman RJ, Miguel JC, Pettigrew JE, Connolly A (2005). Effects of mannan oligosaccharide and an antimicrobial product in nursery diets on performance of pigs reared on three different farms. J Ani Sci..

[32] Duan XD, Chen DW, Zheng P, Tian G, Wang JP, Mao XB, Yu J, He J, Li B, Huang ZQ, Ao ZG, Yu B (2016). Effects of dietary mannan oligosaccharide supplementation on performance and immune response of sows and their offspring. Anim Feed Sci Technol.

[33] Zaloga GP, Siddiqui RA (2004). Biologically active dietary peptides. Mini Rev Med Chem.

[34] Bhat ZF, Kumar S, Bhat HF (2015). Bioactive peptides of animal origin: a review. J Food Sci Technol.

[35] Hou Y, Wu Z, Dai Z, Wang G, Wu G. Protein hydrolysates in animal nutrition: industrial production, bioactive peptides, and functional significance. J Anim Sci Biotechnol. 2017;8:24. 10.1186/s40104-017-0153-9. 10.1186/s40104-017-0153-9 PMC5341468 28286649

[36] Nasri M. Chapter Four - Protein hydrolysates and biopeptides: Production, biological activities, and applications in foods and health benefits. A Review. In: Toldrá F, editor. Advances in food and nutrition research No. 81. Academic Press; 2017. p. 109–59. 10.1016/bs.afnr.2016.10.003. 10.1016/bs.afnr.2016.10.003 28317603

[37] Reddy KV, Yedery RD, Aranha C (2004). Antimicrobial peptides: premises and promises. Int J Antimicrob Agents.

[38] Tang Z, Yin Y, Zhang Y, Huang R, Sun Z, Li T, Chu W, Kong X, Li L, Geng M, Tu Q (2009). Effects of dietary supplementation with an expressed fusion peptide bovine lactoferricin-lactoferrampin on performance, immune function and intestinal mucosal morphology in piglets weaned at age 21 d. Br J Nutr.

[39] Yoon JH, Ingale SL, Kim JS, Kim KH, Lee SH, Park YK, Kwon IK, Chae BJ (2012). Effects of dietary supplementation of antimicrobial peptide-A3 on growth performance, nutrient digestibility, intestinal and fecal microflora and intestinal morphology in weanling pigs. Anim Feed Sci Technol.

[40] Xiao H, Shao F, Wu M, Ren W, Xiong X, Tan B, et al. The application of antimicrobial peptides as growth and health promoters for swine. J Anim Sci Biotechnol. 2015;6:19. 10.1186/s40104-015-0018-z. 10.1186/s40104-015-0018-z PMC4445505 26019864

[41] Cutler SA, Lonergan SM, Cornick N, Johnson AK, Stahl CH (2007). Dietary inclusion of colicin e1 is effective in preventing postweaning diarrhea caused by F18-positive *Escherichia coli* in pigs. Antimicrob Agents Chemother.

[42] Tang X, Fatufe AA, Yin Y, Tang Z, Wang S, Liu Z (2012). Dietary supplementation with recombinant lactoferrampin-lactoferricin improves growth performance and affects serum parameters in piglets. J Anim Vet Adv.

[43] Wu S, Zhang F, Huang Z, Liu H, Xie C, Zhang J, Thacker PA, Qiao S (2012). Effects of the antimicrobial peptide cecropin AD on performance and intestinal health in weaned piglets challenged with *Escherichia coli*. Peptides.

[44] Yoon JH, Ingale SL, Kim JS, Kim KH, Lohakare J, Park YK, Park JC, Kwon IK, Chae BJ (2013). Effects of dietary supplementation with antimicrobial peptide-P5 on growth performance, apparent total tract digestibility, faecal and intestinal microflora and intestinal morphology of weanling pigs. J Sci Food Agric.

[45] Yoon JH, Ingale SL, Kim JS, Kim KH, Lee SH, Park YK, Lee SC, Kwon IK, Chae BJ (2014). Effects of dietary supplementation of synthetic antimicrobial peptide-A3 and P5 on growth performance, apparent total tract digestibility of nutrients, fecal and intestinal microflora and intestinal morphology in weanling pigs. Livest Sci.

[46] Kim J, Nguyen SG, Guevarra RB, Lee I, Unno T (2015). Analysis of swine fecal microbiota at various growth stages. Arch Microbiol.

[47] National Research Council (NRC). Nutrient requirements of swine. Washington: National Academy Press; 2012.

[48] Yu Z, Morrison M (2004). Improved extraction of PCR-quality community DNA from digesta and fecal samples. Biotechniques..

[49] Edwards U, Rogall T, Bloecker H, Emde M, Boettger EC (1989). Isolation and direct complete nucleotide determination of entire genes. Characterization of a gene coding for 16S ribosomal RNA. Nucleic Acids Res.

[50] Lane D, Pace B, Olsen GJ, Stahl DA, Sogin ML, Pace NR (1985). Rapid determination of 16S ribosomal RNA sequences for phylogenetic analyses. Proc Natl Acad Sci U S A.

[51] Poudel P, Levesque CL, Samuel R, St-Pierre B (2020). Dietary inclusion of Peptiva, a peptide-based feed additive, can accelerate the maturation of the fecal bacterial microbiome in weaned pigs. BMC Vet Res.

[52] Schloss PD, Westcott SL, Ryabin T, Hall JR, Hartmann M, Hollister EB, Lesniewski RA, Oakley BB, Parks DH, Robinson CJ, Sahl JW, Stres B, Thallinger GG, van Horn DJ, Weber CF (2009). Introducing mothur: open-source, platform-independent, community-supported software for describing and comparing microbial communities. Appl Environ Microbiol.

[53] Kim M, Morrison M, Yu Z (2011). Evaluation of different partial 16S rRNA gene sequence regions for phylogenetic analysis of microbiomes. J Microbiol Methods.

[54] Kim M, Morrison M, Yu Z (2011). Status of the phylogenetic diversity census of ruminal microbiomes. FEMS Microbiol Ecol.

[55] Johnson JS, Spakowicz DJ, Hong B-Y, Petersen LM, Demkowicz P, Chen L, Leopold SR, Hanson BM, Agresta HO, Gerstein M, Sodergren E, Weinstock GM (2019). Evaluation of 16S rRNA gene sequencing for species and strain-level microbiome analysis. Nat Commun.

[56] Altschul SF, Madden TL, Schäffer AA, Zhang J, Zhang Z, Miller W, Lipman DJ (1997). Gapped BLAST and PSI-BLAST: a new generation of protein database search programs. Nucleic Acids Res.

[57] Wang Q, Garrity GM, Tiedje JM, Cole JR (2007). Naïve bayesian classifier for rapid assignment of rRNA sequences into the new bacterial taxonomy. Appl Environ Microbiol.

[58] Parte AC (2014). LPSN--list of prokaryotic names with standing in nomenclature. Nucleic Acids Res.

[59] Gilbert ER, Wong EA, Webb KEJ (2008). Board-invited review: peptide absorption and utilization: implications for animal nutrition and health. J Ani Sci.

[60] Rerat A, Nunes CS, Mendy F, Roger L (1988). Amino acid absorption and production of pancreatic hormones in non-anaesthetized pigs after duodenal infusions of a milk enzymic hydrolysate or of free amino acids. Br J Nutr.

[61] Kodera T, Hara H, Nishimori Y, Nio N (2006). Amino acid absorption in portal blood after duodenal infusions of a soy protein hydrolysate prepared by a novel soybean protease D3. J Food Sci.

[62] Webb KEJ, Matthews JC, DiRienzo DB (1992). Peptide absorption: a review of current concepts and future perspectives. J Ani Sci..

[63] Daniel H (2004). Molecular and integrative physiology of intestinal peptide transport. Annu Rev Physiol.

[64] Ballou CE (1970). A study of the immunochemistry of three yeast mannans. J Biol Chem.

[65] Ofek I, Mirelman D, Sharon N (1977). Adherence of *Escherichia coli* to human mucosal cells mediated by mannose receptors. Nature..

[66] Spring P, Wenk C, Dawson KA, Newman KE (2000). The effects of dietary mannaoligosaccharides on cecal parameters and the concentrations of enteric bacteria in the ceca of salmonella-challenged broiler chicks. Poult Sci J.

[67] van der Peet-Schwering CM, Jansman AJ, Smidt H, Yoon I (2007). Effects of yeast culture on performance, gut integrity, and blood cell composition of weanling pigs. J Anim Sci.

[68] Castillo M, Martin-Orue SM, Taylor-Pickard JA, Perez JF, Gasa J (2008). Use of mannanoligosaccharides and zinc chelate as growth promoters and diarrhea preventative in weaning pigs: effects on microbiota and gut function. J Anim Sci.

[69] LeMieux FM, Naranjo VD, Bidner TD, Southern LL. Effect of dried brewers yeast on growth performance of nursing and weanling pigs. Prof Anim. 2010;26:70–5. doi: 10.15232/S1080-7446(15)30558-1.

[70] Halas V, Nochta I (2012). Mannan oligosaccharides in nursery pig nutrition and their potential mode of action. Animals..

[71] Farrow JAE, Kruze J, Philips BA, Bramley AJ, Collins MD (1984). Taxonomic studies on *Streptococcus bovis* and *Streptococcus equinus*: description of *streptococcus alactolyticus sp. nov.* and *streptococcus saccharolyticus sp. nov*. Syst Appl Microbiol.

[72] Robinson IM, Stromley JM, Varel VH, Cato EP. *Streptococcus intestinalis*, a new species from the colons and feces of pigs. Int J Syst Evol. 1988;38:245–8. doi: 10.1099/00207713-38-3-245.

[73] Vandamme P, Devriese LA, Haesebrouck F, Kersters K (1999). *Streptococcus intestinalis* Robinson et al. 1988 and *streptococcus alactolyticus* Farrow et al. 1984 are phenotypically indistinguishable. Int J Syst Bacteriol.

[74] Salminen S, Deighton M (1992). Lactic acid bacteria in the gut in normal and disordered states. Dig Dis.

[75] Hudault S, Lievin V, Bernet-Camard MF, Servin AL (1997). Antagonistic activity exerted *in vitro* and *in vivo* by *Lactobacillus casei* (strain GG) against *salmonella typhimurium* C5 infection. Appl Environ Microbiol.

[76] Pascual M, Hugas M, Badiola JI, Monfort JM, Garriga M (1999). *Lactobacillus salivarius* CTC2197 prevents *salmonella enteritidis* colonization in chickens. Appl Environ Microbiol.

[77] Gill HS, Rutherfurd KJ, Prasad J, Gopal PK (2000). Enhancement of natural and acquired immunity by *Lactobacillus rhamnosus* (HN001), *Lactobacillus acidophilus* (HN017) and *Bifidobacterium lactis* (HN019). Br J Nutr.

[78] Vitini E, Alvarez S, Medina M, Medici M, de Budeguer MV, Perdigon G (2000). Gut mucosal immunostimulation by lactic acid bacteria. Biocell..

[79] Kant R, Paulin L, Alatalo E, de Vos WM, Palva A (2011). Genome sequence of *Lactobacillus amylovorus* GRL1118, isolated from pig ileum. J Bacteriol.

[80] Tannock GW, Fuller R, Pedersen K (1990). *Lactobacillus* succession in the piglet digestive tract demonstrated by plasmid profiling. Appl Environ Microbiol.

[81] Guevarra RB, Hong SH, Cho JH, Kim B-R, Shin J, Lee JH, et al. The dynamics of the piglet gut microbiome during the weaning transition in association with health and nutrition. J Anim Sci Biotechnol. 2018;9:54. 10.1186/s40104-018-0269-6. 10.1186/s40104-018-0269-6 PMC6065057 30069307

[82] Guevarra RB, Lee JH, Lee SH, Seok M-J, Kim DW, Kang BN, et al. Piglet gut microbial shifts early in life: causes and effects. J Anim Sci Biotechnol. 2019;10:1. 10.1186/s40104-018-0308-3. 10.1186/s40104-018-0308-3 PMC6330741 30651985

[83] Ortman J, Sinn S, Gibbons W, Brown M, DeRouchey J (2020). St-Pierre B, et al. Comparative analysis of the ileal bacterial composition of post-weaned pigs fed different high-quality protein sources Animal.

[84] Fresno Rueda A, Samuel R, St-Pierre B (2021). Investigating the effects of a phytobiotics-based product on the fecal bacterial microbiome of weaned pigs. Animals..

[85] Naito S, Hayashidani H, Kaneko K, Ogawa M, Benno Y (1995). Development of intestinal *lactobacilli* in normal piglets. J Appl Bacteriol.

[86] Bateup JM, Dobbinson S, Mcconnell MA, Munro K, Tannock GW (1998). Analysis of the composition of *Lactobacillus* populations inhabiting the stomach and caecum of pigs. Microb Ecol Health Dis.

[87] Pieper R, Janczyk P, Zeyner A, Smidt H, Guiard V, Souffrant WB (2008). Ecophysiology of the developing total bacterial and *lactobacillus* communities in the terminal small intestine of weaning piglets. Microb Ecol.

[88] Valeriano VDV, Balolong MP, Kang DK (2017). Probiotic roles of *Lactobacillus* sp. in swine: insights from gut microbiota. J Appl Microbiol.

[89] Nakamura LK. *Lactobacillus amylovorus*, a new starch-hydrolyzing species from cattle waste-corn fermentations. Int J Syst Evol. 1981;31:56–63. doi: 10.1099/00207713-31-1-56.

[90] Åvall-Jääskeläinen S, Palva A (2005). *Lactobacillus* surface layers and their applications. FEMS Microbiol Rev.

[91] Hynonen U, Palva A (2013). *Lactobacillus* surface layer proteins: structure, function and applications. Appl Microbiol Biotechnol.

[92] Taverniti V, Cesari V, Gargari G, Rossi U, Biddau C, Lecchi C, Fiore W, Arioli S, Toschi I, Guglielmetti S (2021). Probiotics modulate mouse gut microbiota and influence intestinal immune and serotonergic gene expression in a site-specific fashion. Front Microbiol.

[93] Pridmore RD, Berger B, Desiere F, Vilanova D, Barretto C, Pittet AC, Zwahlen MC, Rouvet M, Altermann E, Barrangou R, Mollet B, Mercenier A, Klaenhammer T, Arigoni F, Schell MA (2004). The genome sequence of the probiotic intestinal bacterium *Lactobacillus johnsonii* NCC 533. Proc Natl Acad Sci U S A.

[94] Ventura M, Jankovic I, Walker DC, Pridmore RC, Zink R (2002). Identification and characterization of novel surface proteins in *Lactobacillus johnsonii* and *Lactobacillus gasseri*. Appl Environ Microbiol.

[95] Kane MD, Brauman A, Breznak JA (1991). *Clostridium mayombei sp. nov*., an H_2_/CO_2_ acetogenic bacterium from the gut of the African soil-feeding termite, *Cubitermes speciosus*. Arch Microbiol.

[96] Gerritsen J, Fuentes S, Grievink W, van Niftrik L, Tindall BJ, Timmerman HM, Rijkers GT, Smidt H (2014). Characterization of *Romboutsia ilealis gen. Nov*., *sp.nov*., isolated from the gastro-intestinal tract of a rat, and proposal for the reclassification of five closely related members of the genus *Clostridium* into the genera *Romboutsia gen. Nov.*, *Intestinibacter gen. Nov.*, *Terrisporobacter gen. Nov.* and *Asaccharospora gen. Nov*. Int J Syst Bacteriol.

