# Supplementary material for: Investigating the effects of peptide-based, MOS and protease feed additives on the growth performance and fecal microbial composition of weaned pigs
Source: J Anim Sci Biotechnol. 2022 Mar 17;13:25. doi: 10.1186/s40104-022-00681-8 (PMC8928611; doi:10.1186/s40104-022-00681-8)
Supplement: Supplementary file 2 — Additional file 2: Supplementary file 1. Product description for Peptiva, MOS and exogenous protease from the manufacturer (Vitech Bio-Chem Corporation (Glendale, CA, USA). [file 40104_2022_681_MOESM2_ESM.docx]

**Supplementary File 1.** Product description for Peptiva, MOS and exogenous protease from the manufacturer (Vitech Bio-Chem Corporation (Glendale, CA, USA).

**Peptiva**

Peptiva is a peptide-based product generated from a blend of fish, porcine and microbial hydrolysates. Representative chromatographic characteristics and concentrations of the five most abundant peptides are:

| Retention Time (minutes) | Base Peak | Molecular Mass | Volume/Abundance | Relative % Abundance |
| --- | --- | --- | --- | --- |
| 19.648 | 761.94623 | 1521.87822 | 202240 | 1.37% |
| 18.084 | 1092.02345 | 2159.04955 | 163072 | 1.11% |
| 10.413 | 698.35186 | 1394.68916 | 145107 | 0.99% |
| 19.317 | 677.90297 | 1353.79039 | 113591 | 0.77% |
| 14.635 | 1099.5393 | 2196.05797 | 113121 | 0.77% |

**MOS**

The MOS product consists of cell wall and metabolites extracted from cultures of *Saccharomyces cerevisiae.* Its active component is β-1-3, 1-6 glucan.

The concentration of β-1-3, 1-6 glucan is determined by the following method: initial digestion with lyticase in the presence of KOH, then treatment with a mixture of exo-1,3-β-d-glucanase and β-glucosidase to convert β-glucan into glucose.

**Protease**

The protease product, generated from cultures of *Aspergillus Niger*, is provided in powdered form and has a minimum activity of 20,000U/g. One unit of protease activity is defined as the amount of protease product that can release 1 μg of tyrosine per minute from a casein substrate at a temperature of 40℃ and at a pH of 10.5.
